# Survival of nervous system tumors in a Colombian population cancer registry

**DOI:** 10.1007/s11060-025-05313-5

**Published:** 2025-11-17

**Authors:** Constanza Isabel Albarracín Cárdenas, Helio Caballero Rojas, Miguel Enrique Ochoa Vera, María Lucrecia Luna González, Carlos Augusto Rojas Díaz

**Affiliations:** 1 Facultad de Ciencias de la Salud, Universidad Autónoma de Bucaramanga-UNAB, Bucaramanga, Santander Colombia; 2 Registro Poblacional de Cáncer del Área Metropolitana De Bucaramanga, Universidad Autónoma de Bucaramanga-UNAB, Bucaramanga, Santander Colombia

**Keywords:** Primary CNS tumors, Prevalence, Survival, Prognostic factors, Population-based cancer registry

## Abstract

**Background:**

Primary tumors of the central nervous system (CNS) are rare, but they carry high morbidity and mortality. In Latin America, information on survival is limited. This study aimed to identify factors associated with survival of CNS tumors in a Colombian population.

**Methods:**

A retrospective cohort study was conducted with data from the Population Cancer Registry of the Metropolitan Area of Bucaramanga, including cases diagnosed between 2003 and 2017. Sociodemographic and clinical variables were analyzed. Survival was assessed using the Kaplan-Meier method and Cox proportional hazards models. Overall survival at 5 years and hazard ratios (HR) were estimated.

**Results:**

A total of 1126 patients were included, with a median age at diagnosis of 55 years. 60.04% were malignant neoplasms. The most frequent histological subtypes were glioblastoma (27.9% of malignant tumors) and meningioma (80.3% of benign tumors). In total, 67.58% died, with 21.6 years of life lost per patient. Overall survival at 5 years was 57.5%. Malignant tumors had a survival rate of 39.6%. Glioblastoma had the worst prognosis (20.9% survival; HR 9.64; *p* < 0.001). Other significant predictors were: age ≥ 60 years (HR 1.92; *p* < 0.001), male sex (HR 1.37; *p* < 0.001), undifferentiated/anaplastic grade (HR 7.46; *p* < 0.001). No significant association was found between survival and socioeconomic status or type of insurance.

**Conclusion:**

In this cohort, the prognosis of CNS tumors was associated with age, sex, tumor behavior, histological subtype, location, and degree of differentiation. The premature mortality highlights the need to improve diagnostic accuracy and strengthen population registries with clinical and genetic data.

**Supplementary Information:**

The online version contains supplementary material available at 10.1007/s11060-025-05313-5.

## Introduction

Brain and Nervous System (NS) Tumors are rare neoplasms [1–3] but clinically significant, due to their high relative lethality and severe impairment of neurological functions [4–6]. Global reports have reported a progressive increase in the incidence of CNS tumors, driven by population aging and diagnostic advances [4, 7]. Globally, age-adjusted, incidence, and mortality rates of 5.0 and 3.9 per 100,000 inhabitants, respectively, were reported in 2022 [7], In Colombia, according to GLOBOCAN, in 2022, there was an age-adjusted incidence of 4.6 per 100,000 inhabitants, with a mortality of 3.9 per 100,000 [8]. However, data in Colombia on survival are limited, with an information gap especially at the regional level [5, 9, 10], which makes it difficult to accurately characterize public policies or clinical strategies.

CNS tumors comprise a heterogeneous group of neoplasms that affect the brain and spinal cord [5, 11]; they can be classified as primary or secondary, benign, malignant, or uncertain [6]. The current classification according to WHO 2021 incorporates histological, topographic and molecular criteria, especially for gliomas, medulloblastomas and ependymal tumors [12, 13], integrates mutations in genes such as IDH1/2, TP53, ATRX, H3 K27 and G34, and deletions such as 1p/19q or CDKN2A/B, differentiating tumors with different prognoses and treatments despite similar histological morphology [2]. Gliomas represent the most common group among malignant, with glioblastoma as the most common histology, accounting for 46–50.7% of cases [2].

The 5-year relative survival in the USA, according to the CBTRUS, is approximately 91.7% for benign tumors and 34.9% for malignant tumors [2]. There is currently a small number of studies in the country on the survival of these neoplasms in the adult population, including that of Gomez V. et al., who reported overall survival at 6 months and 3 years, of 70.2%, and 35.4%, respectively [9].

Sociodemographic factors such as age over 75 years are associated with lower survival of CNS tumors (HR 13.53; 95% CI 13.06–14.01) [14], male gender (IRR 1.368 95% CI: 1.357–1.378) [15] and having a lower socioeconomic status (HR 1.16; 95%CI1.13-1.18) [14, 16]; tumor characteristics at diagnosis, such as presenting with midline deviation (HR 1.15, *p* = 0.025) [17, 18] and size >3 cm (HR 1.11 95% CI 1.06 1.17) [18]; treatment-related, such as having persistence of residual tumor after extensive surgery (*p* *= 0.001*) [17]. The factors associated with higher survival, sociodemographic, are married marital status (HR 0.90 95% CI 0.84 0.97) [17] and non-Hispanic origin (HR 0.87; 95% CI 0.81–0.93); of the characteristics of the tumor, it is described to be lateralized to the left (HR 0.66 95% CI 0.51–0.85) or to the right (HR 0.65 95% CI 0.50–0.84), adjuvant radiotherapy (HR 0.21 95% CI 0.16–0.27) [19] and gross total tumor resection (HR ref*)* [18].

Population-Based Cancer Registries (PBCRs) are a key source of information on cancer incidence and survival in geographically defined populations. The PBCR in Bucaramanga, Colombia, is one of 460 PBCRs worldwide that provide high-quality data to the International Agency for Research on Cancer (IARC) and the International Association of Cancer Registries (IACR) [20–22].The clinical and histological diversity of CNS tumors, added to the emerging information from PBCRs and international PBCRs (IARC and IACR), constitutes the framework for understanding the factors that influence the incidence, evolution, and prognosis of these tumors in the Colombian population [20, 23–25]. For this reason, this study aimed to determine the factors associated with survival of patients with CNS tumors in the population of the Metropolitan Area of Bucaramanga (AMB).

## Materials and methods

### Study design and data source

A retrospective observational cohort study from a secondary source, collected by the RPC-AMB, was carried out, which included all cases with CNS tumors diagnosed between January 1, 2003 and December 31, 2017, in residents of Bucaramanga, Floridablanca, Girón, and Piedecuesta.

### Case definition and classification

The cases registered by the RPC-AMB met two fundamental requirements, the first, people who resided in the Metropolitan Area of Bucaramanga or resided in the six months prior to the diagnosis of the disease, and the second, that each case had to correspond to a benign or malignant neoplasm of the nervous system. Data on incident cases were actively collected through periodic visits to information sources such as pathology laboratories, imaging, screening, oncology centers, volunteers, medical specialists, hospital discharges, autopsies, and death certificates. These data were systematized through the CanReg5 program, eliminating duplicates, and validated by the IARCTools and Registry PlusTM Link Plus programs [26].

Cases were defined based on the International Classification of Diseases for Oncology, Third Edition (ICD-O-3), including topographies C70.0–C72.9 and C75.1–C75.3 [12]. Tumors were classified as benign, malignant, or of uncertain behavior according to the behavior code in ICD-O-3. Histologic subtypes were grouped according to the 2016 WHO classification of CNS tumors.

Records of soft tissue tumors (fibroblastic and myofibroblastic tumors, vascular tumors, musculoskeletal tumors, and chondroosseous tumors corresponding to morphology codes 8800–8921 and 9120–9176), which despite being anatomically located within the NS, do not derive specifically from NS cells were excluded.

### Study definitions

1. **Location** (primary site): the anatomical site where the neoplasm originated, coded using ICD-O-3 topography.
2. **Grade**: Grade of differentiation describes how closely tumor cells resemble the tissue of origin, use ICD-O-3 as the primary classification for case coding. 1 = well differentiated, 2 = moderately differentiated, 3 = poorly differentiated, 4 = undifferentiated, 9 = unknown.
3. **Histology**: classified by ICD-O-3 morphology and aligned to WHO 2016 CNS groupings.

### Variables and results

Sociodemographic variables included age at diagnosis in years (grouped by decades and by ranges 18–39, 40–59 and over 60 years), sex, municipality of residence (Bucaramanga, Floridablanca, Girón and Piedecuesta, which are the municipalities corresponding to the AMB), socioeconomic status established by the *Departamento Administrativo Nacional de Estadística* (DANE; National Administrative Department of Statistics) and is used primarily to apply differential tariffs for household public utilities (classified into six levels: very low, low, medium low, medium-high, high-low and very high) and health insurance modality (contributory, subsidized, special, prepaid, private, linked or unknown).

Clinical-pathological variables included year of diagnosis, location of the tumor, histological subtype (taking the category of most powerful as a reference); degree of differentiation, behavior (malignant, benign, or uncertain), and life status (living, deceased, or unknown).

### Statistical analysis

Descriptive analyses were performed for all variables. Categorical variables were expressed as absolute and relative frequencies, while continuous variables were summarized using medians and interquartile ranges (IQR) due to non-normal distributions according to the D’Agostino-Royston deviation-kurtosis test. The distribution of the variables was compared by sex and tumor behavior (benign, malignant, uncertain) using the Chi-square test or Fisher’s exact test for categorical variables, and the Mann-Whitney U test for continuous variables. The crude incidence rate of CNS tumors was calculated annually as the number of new cases per 100,000 persons, using the total projected population for the corresponding year from official estimates of the DANE, based on the 2018 National Population and Housing Census (CNPV 2018).

Median overall survival and 5-year survival were calculated using the Kaplan-Meier method, bivariate analyses for survival were performed using the Cox proportional hazards model. To estimate life expectancy, data reported by the WHO, available from cohorts born since 1950, were used [27]. In the case of people born before this date, life expectancy was projected based on the closest available values and the demographic trends observed. The alpha significance level was 0.05. Analyses were performed using STATA version 14 (StataCorp LP, College Station, TX, USA).

This study received endorsement from the Institutional Ethics Committee of the UNAB (CIE-073-2024).

## Results

A total of 1126 records of patients with a de novo diagnosis of a CNS tumor, were analyzed, most of them female (*n* = 636; 56.5%), the median age at diagnosis was 56 years in women and 54 years in men Table 1. The most affected decades were those aged 50–59 years (20.5%) and 60–69 years (18.6%). Women had CNS tumors at younger ages Sup Fig. 1.

58.88% of the patients resided in Bucaramanga. The socioeconomic classes “lower middle” (35.4%) and “low high” (27.7%) were the most frequent. As for insurance, 64.9% were affiliated to the contributory regime, followed by the subsidized (20.4%).

### Characteristics of the population according to sex

Bucaramanga was the municipality with the highest proportion of cases in both sexes (56.6% women; 55.9% men), although Floridablanca had a higher proportion of women (36% vs. 21.2%). This difference was statistically significant only for Bucaramanga (*p* = 0.027). Regarding socioeconomic status, a higher proportion of men was found in the “low low” stratum (11.9%) and more women in the “high high” stratum (4.6% vs. 1.3%), with significant overall differences (*p* = 0.02). Regarding insurance, the contributory regime was predominant in both sexes (65.6% women vs. 64.1% men), with no significant differences (*p* = 0.347), Table 1.

**Table 1:** Sociodemographic variables by sex

Characteristics	Total *n* = 1126 (100%) *n* (%)	Female *n* = 636 (56.48%) *n* (%)	Male *n* = 490 (43.52%) *n* (%)	*p*-value
**Age (years)**,** median (IQR)**	55 (42–67)	56 (42–67)	54 (41–67)	0.086*
**Place of residence**				
Bucaramanga	663 (58.88)	383 (56.6)	274 (55.92)	0.027†
Floridablanca	249 (22.11)	223 (36)	104 (21.22)	
Piedecuesta	115 (10.21)	58 (9.12)	57 (11.63)	
Girón	99 (8.79)	44 (6.92)	55 (11.22)	
**Socioeconomic status**				
Low bass	88 (10.07)	41 (8.58)	47 (11.87)	0.02†
Low high	242 (27.69)	124 (25.94)	118 (29.8)	
Medium Low	309 (35.35)	173 (36.19)	136 (34.34)	
Medium High	174 (19.91)	102 (21.34)	72 (18.18)	
High Low	34 (3.89)	16 (3.35)	18 (4.55)	
High High	27 (3.09)	22 (4.6)	5 (1.26)	
**Health insurance modality**				
Contributory	731 (64.92)	417 (65.57)	314 (64.08)	0.347‡
Subsidized	230 (20.43)	122 (19.18)	108 (22.04)	
Special	98 (8.7)	63 (9.91)	35 (7.14)	
Unknown	40 (3.55)	20 (3.14)	20 (4.08)	
Prepaid Medicine	22 (1.95)	12 (1.89)	10 (2.04)	
Linked	4 (0.36)	1 (0.16)	3 (0.61)	
Particular	1 (0.09)	1 (0.16)	0 (0)	

* Mann Whitney test, † χ² test, ‡ Fisher’s test

Cerebral meninges were the most common location (24.25%), with a predominance in women (32.23% vs. 13.88%), while brain and overlapping tumors were more frequent in men (56.82% and 53.75%, respectively). Women had more benign tumors (40.41%), and men had more undifferentiated/anaplastic tumors (25.10%). Histologically, meningiomas (in all its variants, including the transitional type) were more frequent in women, while glioblastomas, astrocytomas, and nonspecific malignancies predominated in men. More ependymomas and hemangioblastomas were also observed in males. The differences by histological subtype according to sex were statistically significant (*p* < 0.001). No differences were identified in the distribution by year of diagnosis (*p* = 0.341), but there were differences in vital status, location, degree of differentiation and histological type (*p* < 0.001 in all cases) Table 2.

**Table 2:** Clinical-pathological variables according to sex

Characteristics	Total *n* = 1126 (100%) *n* (%)	Female *n* = 636 (56.48%) *n* (%)	Male *n* = 490 (43.52%) *n* (%)	*p*-value
**Tumor location**				
Cerebral meninges	273 (24.25)	205 (32.23)	68 (13.88)	< 0.001‡
Encephalon, SAI	176 (15.63)	76 (11.95)	100 (20.41)	
Brain, SAI	160 (14.21)	79 (12.42)	81 (16.53)	
Frontal lobe	84 (7.46)	44 (6.92)	40 (8.16)	
Overlapping lesion of contiguous sites of the brain	80 (7.10)	37 (5.82)	43 (8.78)	
Temporal lobe	72 (6.39)	34 (5.35)	38 (7.76)	
Pituitary gland	59 (5.24)	25 (3.93)	34 1 (6.94)	
Meninges, SAI	48 (4.26)	36 (5.66)	12 (2.45)	
Parietal lobe	40 (3.55)	26 (4.09)	14 (2.86)	
Cerebellum SAI	36 (3.20)	19 (2.99)	17 (3.47)	
Lóbulo occipital	21 (1.87)	10 (1.57)	11 (2.24)	
Spinal meninges	17 (1.51)	14 (2.20)	3 1 (0.61)	
Spinal cord	13 (1.15)	6 (0.94)	7 (1.43)	
nervous system SAI	13 (1.15)	7 (1.10)	6 (1.22)	
Ventricle, SAI	12 (1.07)	15 (0.79)	7 (1.43)	
Craniopharyngeal Duct	12 (1.07)	7 (1.10)	5 (1.02)	
Brain stem	5 (0.44)	3 (0.47)	2 (0.41)	
Auditory nerve	2 (0.18)	1 (0.16)	1 (0.20)	
Pineal body	2 (0.18)	2 (0.31)	0 (0.00)	
Overlapping lesion of contiguous sites of the CNS	1 (0.09)	0 (0.00)	1 (0.20)	
**Degree of Differentiation**				
Unknown	420 (37.30)	211 (33.18)	209 (42.65)	
Benign	360 (31.97)	257 (40.41)	103 (21.02)	
Undifferentiated/Anaplastic	235 (20.87)	112 (17.61)	123 (25.10)	
Moderately differentiated	43 (3.82)	20 (3.14)	23 (4.69)	
Poorly Difference	32 (2.84)	17 (2.67)	15 (3.06)	
Well differentiating	23 (2.04)	11 (1.73)	12 (2.45)	
Cell B	13 (1.15)	8 (1.26)	5 (1.02)	
**Histological Subtype**				
Neoplasia maligna	192 (17.05)	92 (14.47)	100 (20.41)	
Glioblastoma, NOS	184 (16.34)	86 (13.52)	98 (20.00)	
Meningothelial meningioma	109 (9.68)	81 (12.74)	28 (5.71)	
Malignant Meningioma	91 (8.08)	63 (9.91)	28 (5.71)	
Astrocytoma, NOS	82 (7.28)	40 (6.29)	42 (8.57)	< 0.001†
Anaplastic astrocytoma	65 (5.77)	36 (5.66)	29 (5.92)	
Pituitary carcinoma	55 (4.88)	23 (3.62)	32 (6.53)	
Fibrous meningioma	47 (4.17)	37 (5.82)	10 (2.04)	
Transitional meningioma	34 (3.02)	28 (4.40)	6 (1.22)	
Fibrillar astrocytoma	26 (2.31)	12 (1.89)	14 (2.86)	
Psammomatous meningioma	25 (2.22)	22 (3.46)	3 (0.61)	
Other♦				

† χ² test ‡ Fisher’s test

♦ Other: Sup Table 1

### Characteristics of the population according to tumor behavior

Tumor behavior was malignant in 60.04%, benign in 31.97%, and uncertain in 7.99%. Malignant tumors were slightly more frequent in men (51.92% vs. 48.08%), but when comparing the proportions, men had a higher number of malignant tumors (71.6%) than benign tumors (21%) (*p* < 0.001). Benign tumors predominated in women (71.39% vs. 48.08%) *Sup Table 2*.

The most common locations were the cerebral meninges (24.25%) and the brain (15.63%). Meningeal lesions had a predominant proportion of benign neoplasms (86.1%), while encephalic lesions were mostly malignant (83.5%). Most cases were diagnosed by histology of the primary tumor (80.02%), with a predominance of malignant tumors (56.94%). Uncertain tumors are most commonly diagnosed by clinical procedures (17.39%) and death certificates (13.16%). Most malignant tumors were undifferentiated/anaplastic (34.76% of the total) and to a lesser extent B-cell and poorly differentiated Table 3.

Histologically, astrocytomas and glioblastoma were the most common malignant tumors (approximately 27.9% of all malignant tumors). In benign tumors, meningiomas accounted for 80.3%, especially meningotelial (30.28%), and relevant frequencies of other neoplasms such as pituitary adenoma, schwannoma and pilocytic astrocytoma were also observed. In tumors of uncertain behavior, meningeal sarcomatosis (14.4%), hemangioblastoma (18.9%), and craniopharyngioma (11.1%) stood out. 17% of records lacked specific histology.

**Table 3:** Clinical-pathological variables according to tumor behavior

Characteristics	Total *n* = 1126 (100%) *n* (%)	Malignant *n* = 676 (60.04%) *n* (%)	Benign *n* = 360 (31.97%) *n* (%)	Uncertain *n* = 90 (7.99%) *n* (%)	*p*-value
**Tumor location**					- < 0.001‡
Cerebral meninges	273 (24.25)	18 (2.66)	235 (65.28)	20 (22.22)	
Encephalon, SAI	176 (15.63)	147 (21.75)	4 (1.11)	25 (27.78)	
Brain, SAI	160 (14.21)	150 (22.19)	0 (0.00)	10 (11.11)	
Frontal lobe	84 (7.46)	83 (12.28)	0 (0.00)	1 (1.11)	
Overlapping lesion of contiguous sites of the brain	80 (7.10)	79 (11.69)	0 (0.00)	1 (1.11)	
Temporal lobe	72 (6.39)	71 (10.50)	0 (0.00)	1 (1.11)	
Pituitary gland	59 (5.24)	4 (0.59)	54 (15.00)	1 (1.11)	
Meninges, SAI	48 (4.26)	7 (1.04)	39 (10.83)	2 (2.22)	
Parietal lobe	40 (3.55)	39 (5.77)	0 (0.00)	1 (1.11)	
Cerebellum SAI	36 (3.20)	24 (3.55)	4 (1.11)	8 (8.89)	
Lóbulo occipital	21 (1.87)	19 (2.81)	0 (0.00)	2 (2.22)	
Spinal meninges	17 (1.51)	0 (0.00)	16 (4.44)	1 (1.11)	
Spinal cord	13 (1.15)	10 (1.48)	2 (0.56)	1 (1.11)	
nervous system SAI	13 (1.15)	10 (1.48)	3 (0.83)	1 (0.00)	
Ventricle, SAI	12 (1.07)	7 (1.04)	1 (0.28)	4 (4.44)	
Craniopharyngeal Duct	12 (1.07)	0 (0.00)	0 (0.00)	12 (13.33)	
Brain stem	5 (0.44)	5 (0.74)	0 (0.00)	0 (0.00)	
Auditory nerve	2 (0.18)	0 (0.00)	2 (0.56)	0 (0.00)	
Pineal body	2 (0.18)	2 (0.30)	0 (0.00)	0 (0.00)	
Overlapping lesion of contiguous sites of the CNS	1 (0.09)	1 (0.15)	0 (0.00)	0 (0.00)	
**Diagnostic Method**					
Histology Primary Tumor	901 (80.02)	513 (75.89)	330 (91.67)	58 (64.44)	< 0.001
Death Certificate	76 (6.75)	60 (8.88)	6 (1.67)	10 (11.11)	
Clinical Only	76 (6.75)	60 (8.88)	6 (1.67)	10 (11.11)	
Clinical Procedures	69 (6.13)	40 (5.92)	17 (4.72)	12 (13.33)	
Cytology or Hematology	3 (0.27)	2 (0.30)	1 (0.28)	0 (0.00)	
Metastasis Histology	1 (0.09)	1 (0.15)	0 (0.00)	0 (0.00)	
**Degree of Differentiation**					
Unknown	420 (37.30)	330 (78.57)	360 (99)	90 (48.82)	< 0.001†
Benign	360 (31.97)	0 (0.00)	0 (0)	0 (0)	
Undifferentiated/Anaplastic	235 (20.87)	235 (34.76)	0 (0)	0 (0)	
Moderately differentiated	43 (3.82)	43 (6.36)	0 (0)	0 (0)	
Poorly Difference	32 (2.84)	32 (4.73)	0 (0)	0 (0)	
Well differentiating	23 (2.04)	23 (3.40)	0 (0)	0 (0)	
Cell B	13 (1.15)	13 (1.92)	5 (1.02)	0 (0)	
**Histological Subtype**					
Neoplasia maligna	192 (17.05)	158 (23.37)	3 (0.83)	31 (34.44)	< 0.001†
Glioblastoma, NOS	184 (16.34)	184 (27.22)	0 (0.00)	0 (0.00)	
Meningioma meningotelial	109 (9.68)	0 (0.00)	109 (30.28)	0 (0.00)	
Astrocytoma, NOS	82 (7.28)	82 (12.13)	0 (0.00)	0 (0.00)	
Meningioma	71 (6.30)	0 (0.00)	65 (18.06)	6 (6.67)	
Anaplastic astrocytoma	65 (5.77)	65 (9.62)	0 (0.00)	0 (0.00)	
Pituitary adenoma	53 (4.70)	0 (0.00)	53 (14.72)	0 (0.00)	
Fibrous meningioma	47 (4.17)	0 (0.00)	47 (13.06)	0 (0.00)	
Transitional meningioma	34 (3.02)	0 (0.00)	34 (9.44)	0 (0.00)	
Fibrillar astrocytoma	26 (2.31)	26 (3.85)	0 (0.00)	0 (0.00)	
Meningioma psamomatoso	25 (2.22)	0 (0.00)	25 (6.94)	0 (0.00)	
Malignant Meningioma	20 (1.77)	20 (2.96)	0 (0.00)	0 (0.00)	
Other♦					

† χ² test ‡ Fisher’s test

♦ Other: Sup Table 3

An incidence of 7.16 cases per 100,000 person-years and a mortality of 4 deaths per 100,000 person-years were evidenced for the period from 2003 to 2017 Fig. 1.

**Fig. 1:**
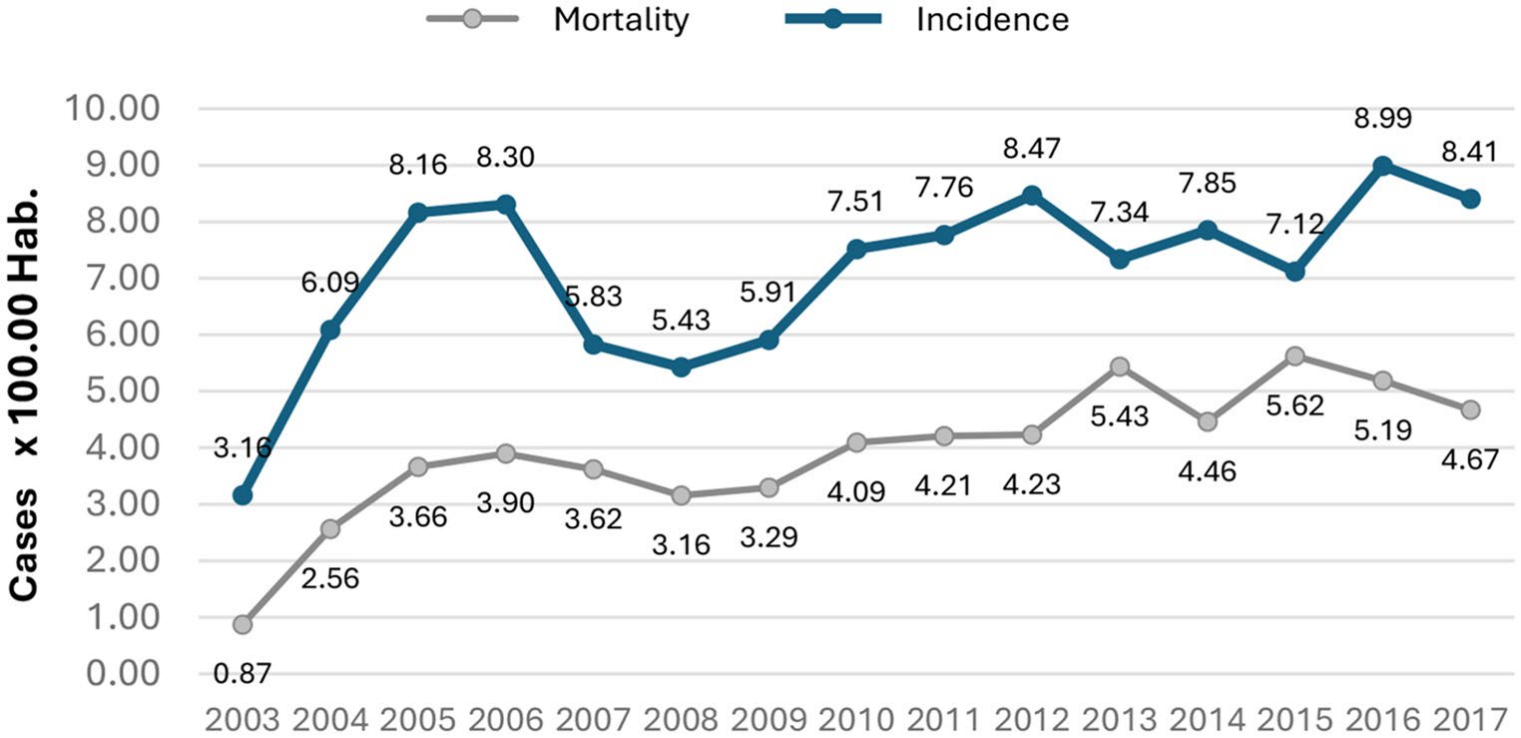
Incidence and Mortality, 2003 to 2017

The early rise in incidence (2003–2006) is consistent with registry maturation and consolidation of reporting sources. The subsequent dip (2007–2009) coincided with system-wide access disruptions in Colombia’s health system—differences by insurance regime and widespread service denials—prompting Constitutional Court Judgment C-760/2008 and benefits-plan equalization from 2010. These system-level dynamics likely reduced case ascertainment transiently, contributing to the observed variability.

As of the date of update of the vital status (November 2024), 67.58% of the patients in the registry had died, of which 75.56% had a diagnosis of a malignancy; on the other hand, living patients (31.97%) had a higher number of benign tumors (63.89%). Although more women died 52 vs. 47%, proportionally a higher percentage of men die compared to the total of them. (74.49% of men and 62.26% of women died *Sup Table 4*.

**Fig. 2:**
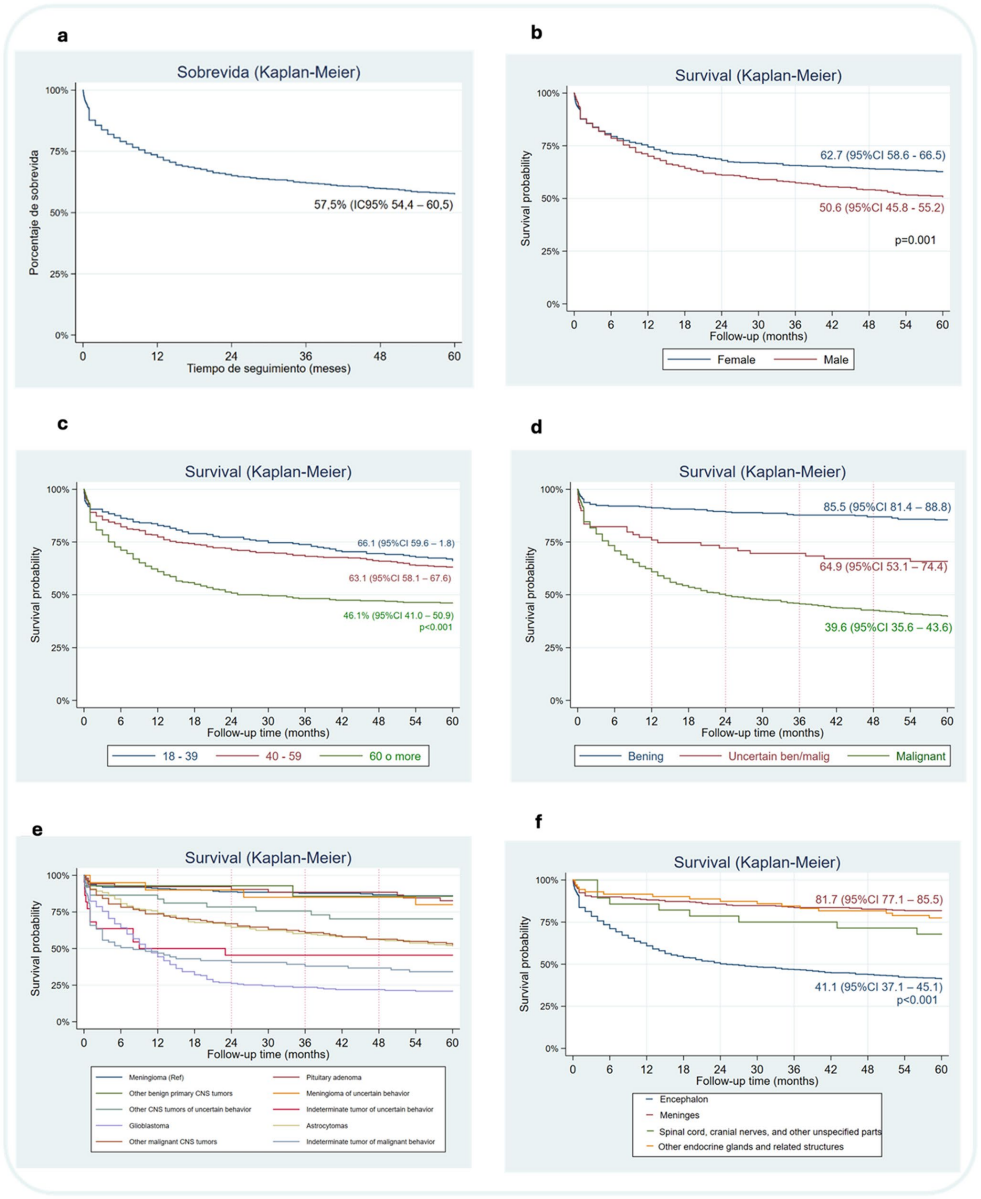
Kaplan-Meier curves of 5-year survival. **a** Overall survival of CNS tumors **b** Survival by sex, **c** Survival by age, **d** Survival by tumor behavior, **e** Survival by histological subtype, **f** Survival by location of the CNS tumor

In the survival analysis, the overall 5-year rate was 57.5% The CNS tumors generated a total of 6,380 years of life lost (YLL) among 295 patients, i.e. 21.6 YLLs for each patient Fig. 2a.

Women showed better survival (62.7%) than men (50.6%) (*p* = 0.001) Fig. 2b. Being over 60 years of age at the time of diagnosis was associated with a survival rate of 46.09% Fig. 2c. Malignant tumors had a 5-year survival rate of 39.6%, compared with 85.5% for benign tumors Fig. 2d. Glioblastoma had the worst survival (20.9%), while benign meningiomas reached 86.1% Fig. 2e. The location was also significant, brain tumors had lower survival (41.1%) compared to meninges tumors (81.7%) (*p* < 0.001) Fig. 2f. The survival for each of the variables of the study are detailed in the *Sup Table 5*.

Poor prognostic factors were identified as age over 60 years (HR: 1.92; 95% CI: 1.48–2.49), male (HR: 1.37; 95% CI: 1.14–1.66), having an uncertain or malignant neoplasm (HR 2.85, 95% CI 1.78–4.54 and HR 5.74, 95% CI 4.28–7.71), Degree of differentiation type Undifferentiated/Anaplastic and B cells (HR 7.46, 95% CI 5.43–10.23 and HR 7.86, 95% CI 3.86–15.98), having a brain location (Ref.), diagnosis of glioblastoma (HR: 9.64 95% CI 6.78–13.72), Indeterminate tumor of malignant behavior (HR 7.98 95% CI 5.28–12.06), Indeterminate tumor of behavior Uncertain (HR 6.08, 95% CI 3.19–11.6) Astrocytomas (HR 4.14 95% CI: 2.85–6.014).

The summary of the factors found to be associated with overall survival and their respective measures of association are shown in the *Sup Fig. 2.*

## Discussion

This study provides a comprehensive analysis of the demographic and clinical-pathological factors associated with the behavior and prognosis of CNS tumor in AMB.

The geographic analysis showed disparities in the distribution and outcome of the NDCs, with a higher proportion of malignancy in municipalities other than Bucaramanga (capital of the region), which could reflect inequities in access to specialized diagnosis and treatment. Age over 60 years and male sex were associated with worse survival, confirming trends previously described in the international literature [3, 15, 28, 29], although with an earlier risk threshold in our population. Although women had a higher frequency of CNS tumors, men concentrated malignant tumors and had lower survival rates.

Although previous studies have explored the relationship between socioeconomic and insurance factors with the survival of CNS tumors [9, 14, 24, 30], no significant association was found in this cohort, suggesting that other clinical variables have greater prognostic weight [31].

Clinicopathological features with relevant findings include high glioblastoma-associated mortality, with an HR close to 10 (HR 9,64, IC95% 6,78 − 13,72), followed by indeterminate tumors of malignant and uncertain behavior (HR 7.98, 95% CI 5.28–12.06 and HR 6.08, 95% CI 3.19–11.6, respectively) and astrocytomas 4.14 (2.85–6.014); the clear relationship between brain location and adverse prognosis, in contrast to meningeal or cranial nerve tumors, of better survival at 5 years. Likewise, the degree of tumor differentiation proved to be a strong predictor of survival, with undifferentiated/anaplastic and B-cell patients having a 7-fold higher risk of death compared to the T-cell group (HR 7.46 95% CI: 5.43–10.23 and HR 7.86 95% CI: 3.86–15.98 respectively); reinforcing the importance of histological characterization. The increasing proportion of tumors with uncertain behavior suggests possible deficiencies in diagnostic characterization in recent years, which manifests the need to include information on surgical management [18], neuroimaging findings and molecular/genetic alterations, which allow strengthening the traceability of CNS tumors in population registries, and thus contribute to the improvement in care, equity and prognosis of patients. In this context, the lack of routine molecular data (e.g., IDH) during 2003–2017 may have inflated survival estimates for histology-defined “glioblastoma” by pooling cases that, under WHO 2021, would be classified as astrocytoma, IDH-mutant, grade 4, which typically show longer survival than glioblastoma, IDH-wildtype.

Publications are available in the scientific literature that offer valuable information on the incidence and general survival of CNS tumors [15, 28]; however, few studies address survival risk relationships between histological subtypes [14], . In contrast, in this cohort, although clusters based on biological behavior (benign, malignant, and uncertain) were used, it was possible to estimate differentiated risk ratios (HRs) for several subtypes within these categories, which allowed for a more detailed prognostic analysis than that reported in other studies, providing more accurate local evidence and underscoring the relevance of this research.

## Conclusions

The results of this study confirm that advanced age, male sex, histological subtypes such as glioblastoma, an indeterminate tumor of malignant and uncertain behavior, astrocytomas, meningiomas of uncertain behavior and location at the brain level (cerebral lobes, basal nuclei, thalamus and corpus callosum), are key determinants in the survival of patients with CNS tumors from AMB. Unlike some previous studies, there was no association between socioeconomic class or insurance modality and clinical outcome, suggesting that clinical factors have a greater impact on this population. The high burden of premature mortality associated with CNS tumors highlighted, generating 21.6 YLLs for each patient.

This work highlights the need to improve diagnostic accuracy, including information on surgical management, neuroimaging findings, genetic alterations, and to promote research with multivariate models adjusted for tumor subtype.

## Supplementary Information

Below is the link to the electronic supplementary material.

Supplementary Material 1

## Data Availability

The data used in this study were obtained from the population-based cancer registry of the Metropolitan Area of Bucaramanga (RPC-AMB), Colombia. These data are not publicly available due to institutional and ethical restrictions but may be made available upon reasonable request and with permission from the data owner. Researchers interested in accessing the data may contact the registry through cancerbmanga@unab.edu.co.
