# Supplementary material for: Survival of nervous system tumors in a Colombian population cancer registry
Source: J Neurooncol. 2025 Nov 17;176(1):84. doi: 10.1007/s11060-025-05313-5 (PMC12628375; doi:10.1007/s11060-025-05313-5)

**Supplementary Material**

**Sup Figure 1
Age at diagnosis of SID, by sex**


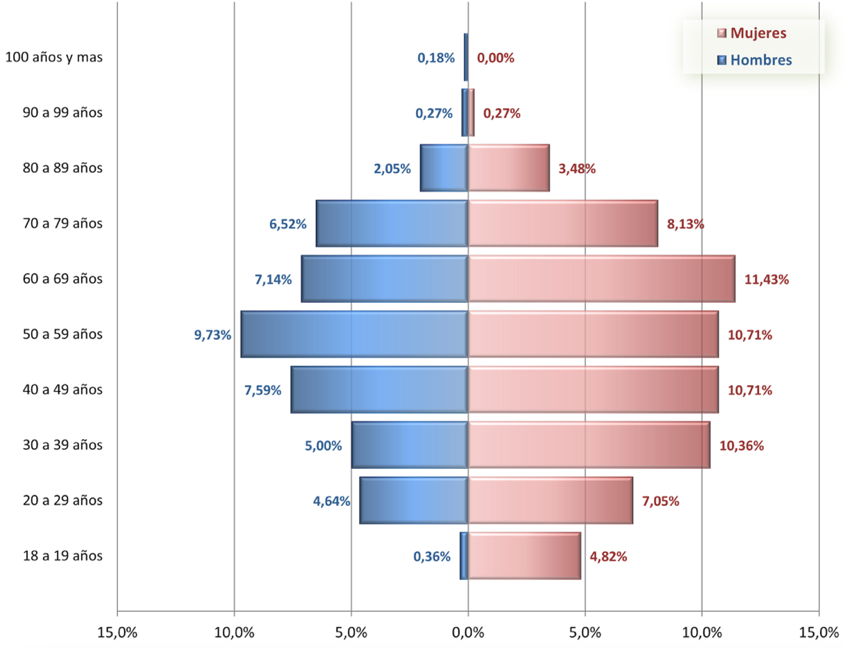


**Sup table 1. Histological subtype according to sex**

Neoplasms with fewer than 20 records per histological subtype

| **Characteristics** | **Total n=1126 (100%)** | **Female n=636 (56,48%)** | **Male n=490 (43,52%)** | **p-value** |
| --- | --- | --- | --- | --- |
| **Histological Subtype** |  |  |  |  |
| Oligodendroglioma, NOS | 19 (1,69) | 9 (1,42) | 10 (2,04) |  |
| Hemangioblastoma | 17 (1,51) | 7 (1,10) | 10 (2,04) |  |
| Malignant glioma | 17 (1,51) | 6 (0,94) | 11 (2,24) |  |
| Ependymoma, NOS | 16 (1,42) | 6 (0,94) | 10 (2,04) |  |
| Gemistocytic Astrocytoma | 15 (1,33) | 6 (0,94) | 9 (1,84) |  |
| Meningeal Sarcomatosis | 14 (1,24) | 12 (1,89) | 2 (0,41) |  |
| Malignant neurilemoma | 13 (1,15) | 8 (1,26) | 5 (1,02) |  |
| Malignant B-cell lymphoma largas difuso (NO) | 12 (1,07) | 7 (1,10) | 5 (1,02) |  |
| Craneopharyngioma | 10 (0,89) | 7 (1,10) | 3 (0,61) |  |
| Mixed glioma | 9 (0,80) | 5 (0,79) | 41 (0,82) |  |
| Medulloblastoma, NOS | 9 (0,80) | 5 (0,79) | 4 (0,82) |  |
| Anaplastic oligodendroglioma | 8 (0,71) | 3 (0,47) | 5 (1,02) |  |
| Angiomatous meningioma | 8 (0,71) | 7 (1,10) | 1 (0,20) |  |
| Pilocytic astrocytoma | 5 (0,44) | 4 (0,63) | 1 (0,20) | < 0,001† |
| Giant cell glioblastoma | 5 (0,44) | 5 (0,79) | 0 (0,00) |  |
| Malignant lymphoma (NOS) | 5 (0,44) | 3 (0,47) | 2 (0,41) |  |
| Choroid plexus carcinoma | 3 (0,27) | 2 (0,31) | 1 (0,20) |  |
| Gliosarcoma | 3 (0,27) | 1 (0,16) | 2 (0,41) |  |
| Subependymoma | 2 (0,18) | 1 (0,16) | 1 (0,20) |  |
| Papillary mixed ependymoma | 2 (0,18) | 1 (0,16) | 1 (0,20) |  |
| Neurocytoma | 2 (0,18) | 2 (0,31) | 0 (0,00) |  |
| Papillary meningioma | 2 (0,18) | 2 (0,31) | 0 (0,00) |  |
| Non-Hodgkin malignant lymphoma | 2 (0,18) | 0 (0,00) | 2 (0,41) |  |
| Extramedullary plasmacytoma | 2 (0,18) | 1 (0,16) | 1 (0,20) |  |
| Prolactinoma | 1 (0,09) | 0 (0,00) | 1 (0,20) |  |
| Meningeal melanomatosis | 1 (0,09) | 0 (0,00) | 1 (0,20) |  |
| Teratoma maligno (NOS) | 1 (0,09) | 0 (0,00) | 1 (0,20) |  |
| Teratoma with transformation | 1 (0,09) | 1 (0,16) | 0 (0,00) |  |
| Adamantinomatous craniopharyngioma | 1 (0,09) | 0 (0,00) | 1 (0,20) |  |
| Papillary craniopharyngioma | 1 (0,09) | 0 (0,00) | 1 (0,20) |  |
| Chordoma, NOS | 1 (0,09) | 1 (0,16) | 0 (0,00) |  |
| Chordoid chordoma | 1 (0,09) | 0 (0,00) | 1 (0,20) |  |
| Papillary tumor of the pineal region | 1 (0,09) | 1 (0,16) | 0 (0,00) |  |
| Protoplasmic astrocytoma | 1 (0,09) | 0 (0,00) | 1 (0,20) |  |
| Primitive neuroectodermal tumor | 1 (0,09) | 0 (0,00) | 1 (0,20) |  |
| Neuroblastoma, NOS | 1 (0,09) | 1 (0,16) | 0 (0,00) |  |
| Anaplastic ganglioglioma | 1 (0,09) | 0,00 () | 1 (0,20) |  |
| Hemangioblastic meningioma | 1 (0,09) | 1 (0,16) | 0 (0,00) |  |
| Malignant peripheral nerve sheath tumor | 1 (0,09) | 0,00 () | 1 (0,20) |  |
| Hodgkin lymphoma with nodular sclerosis | 1 (0,09) | 1 (0,16) | 0 (0,00) |  |

† χ² test

Sup table 2
Sociodemographic variables according to tumor behavior

| **Characteristics** | **Total n=1126 (100%)n (%)** | **Malignant n=676 (60,04%)**  **n (%)** | **Benign n=360 (31,97%)**  **n (%)** | **Uncertain n=90 (7,99%)**  **n (%)** | **p-value** |
| --- | --- | --- | --- | --- | --- |
| **Sex** |  |  |  |  |  |
| Female | 636 (56.48) | 325 (48.08) | 257 (71.39) | 54 (60.00) | <0,001* |
| Male | 490 (43.52) | 351 (51.92) | 103 (28.61) | 36 (40.00) |  |
| **Place of residence** |  |  |  |  |  |
| Bucaramanga | 663 (58,88) | 383 (56.66) | 234 (65.00) | 46 (51.11) | 0.011* |
| Floridablanca | 249 (22,11) | 144 (21.30) | 78 (21.67) | 27 (30.00) |  |
| Piedecuesta | 115 (10,21) | 79 (11.69) | 28 (7.78) | 8 (8.89) |  |
| Girón | 99 (8,79) | 70 (10.36) | 20 (5.56) | 9 (10.00) |  |
| **Socioeconomic status** |  |  |  |  |  |
| Low bass | 88 (10,07) | 53 (8.94) | 24 (11.06) | 11 (17.19) | 0,203† |
| Low high | 242 (27,69) | 167 (28.16) | 55 (25.35) | 20 (31.25) |  |
| Medium Low | 309 (35,35) | 219 (36.93) | 70 (32.26) | 20 (31.25) |  |
| Medium High | 174 (19,91) | 114 (19.22) | 52 (23.96) | 8 (12.50) |  |
| High Low | 34 (3,89) | 23 (3.88) | 10 (4.61) | 1 (1.56) |  |
| High High | 27 (3,09) | 17 (2.87) | 6 (2.76) | 4 (6.25) |  |
| **Health insurance modality** |  |  |  |  |  |
| Contributory | 731 (64,92) | 435 (64.35) | 246 (68.33) | 50 (55.56) | 0,258† |
| Subsidized | 230 (20,43) | 150 (22.19) | 58 (16.11) | 22 (24.44) |  |
| Special | 98 (8,7) | 52 (7.69) | 35 (9.72) | 11 (12.22) |  |
| Unknown | 40 (3,55) | 26 (3.85) | 10 (2.78) | 4 (4.44) |  |
| Prepaid Medicine | 22 (1,95) | 10 (1.48) | 10 (2.78) | 2 (2.22) |  |
| Linked | 4 (0,36) | 2 (0.30) | 1 (0.28) | 1 (1.11) |  |
| Particular | 1 (0,09) | 1 (0.15) | 0 (0.00) | 0 (0.00) |  |

* χ² test, † Fisher test

Sup table 3
**Histological subtype according to tumor behavior**

Neoplasms with fewer than 20 records per histological subtype

| **Characteristics** | **Total n=1126**  **(100%)** | **Malignant n=676**  **(60,04%)** | **Benign n=360**  **(31,97%)** | **Uncertain n=90**  **(7,99%)** | **p-value** |
| --- | --- | --- | --- | --- | --- |
| **Histological Subtype** |  |  |  |  |  |
| Oligodendroglioma, NOS | 19 (1,69) | 19 (2,81) | 0 (0,00) | 0 (0,00) |  |
| Hemangioblastoma | 17 (1,51) | 0 (0,00) | 0 (0,00) | 17 (18,89) |  |
| Malignant glioma | 17 (1,51) | 17 (2,51) | 0 (0,00) | 0 (0,00) |  |
| Ependimoma, NOS | 16 (1,42) | 16 (2,37) | 0 (0,00) | 0 (0,00) |  |
| Astrocytoma Gemistocítico | 15 (1,33) | 15 (2,22) | 0 (0,00) | 0 (0,00) |  |
| Meningeal Sarcomatosis | 14 (1,24) | 0 (0,00) | 1 (0,28) | 13 (14,44) |  |
| Malignant neurilemoma | 13 (1,15) | 1 (0,15) | 11 (3,06) | 1 (1,11) |  |
| Malignant lymphoma diffuse long B cells (NO) | 12 (1,07) | 12 (1,78) | 0 (0,00) | 0 (0,00) |  |
| Craneopharyngioma | 10 (0,89) | 0 (0,00) | 0 (0,00) | 10 (11,11) |  |
| Mixed glioma | 9 (0,80) | 9 (1,33) | 0 (0,00) | 0 (0,00) | < 0,001† |
| Medulloblastoma, NOS | 9 (0,80) | 9 (1,33) | 0 (0,00) | 0 (0,00) |  |
| Anaplastic oligodendroglioma | 8 (0,71) | 8 (1,18) | 0 (0,00) | 0 (0,00) |  |
| Angiomatous meningioma | 8 (0,71) | 0 (0,00) | 8 (2,22) | 0 (0,00) |  |
| Pilocytic astrocytoma | 5 (0,44) | 0 (0,00) | 0 (0,00) | 5 (5,56) |  |
| Giant cell glioblastoma | 5 (0,44) | 5 (0,74) | 0 (0,00) | 0 (0,00) |  |
| Malignant lymphoma (NOS) | 5 (0,44) | 5 (0,74) | 0 (0,00) | 0 (0,00) |  |
| Choroid plexus carcinoma | 3 (0,27) | 2 (0,30) | 1 (0,28) | 0 (0,00) |  |
| Gliosarcoma | 3 (0,27) | 3 (0,44) | 0 (0,00) | 0 (0,00) |  |
| Subependymoma | 2 (0,18) | 1 (0,15) | 0 (0,00) | 1 (1,11) |  |
| Papillary mixed ependymoma | 2 (0,18) | 2 (0,30) | 0 (0,00) | 0 (0,00) |  |
| Pituitary carcinoma | 2 (0,18) | 2 (0,30) | 0 (0,00) | 0 (0,00) |  |
| Neurocytoma | 2 (0,18) | 0 (0,00) | 0 (0,00) | 2 (2,22) |  |
| Papillary meningioma | 2 (0,18) | 1 (0,15) | 0 (0,00) | 1 (1,11) |  |
| Non-Hodgkin malignant lymphoma | 2 (0,18) | 2 (0,30) | 0 (0,00) | 0 (0,00) |  |
| Extramedullary plasmacytoma | 2 (0,18) | 2 (0,30) | 0 (0,00) | 0 (0,00) |  |
| Prolactinoma | 1 (0,09) | 0 (0,00) | 1 (0,28) | 0 (0,00) |  |
| Meningeal melanomatosis | 1 (0,09) | 0 (0,00) | 0 (0,00) | 1 (1,11) |  |
| Malignant teratoma (NOS) | 1 (0,09) | 1 (0,15) | 0 (0,00) | 0 (0,00) |  |
| Teratoma with transformation | 1 (0,09) | 0 (0,00) | 1 (0,28) | 0 (0,00) |  |
| Adamantinomatous craniopharyngioma | 1 (0,09) | 0 (0,00) | 0 (0,00) | 1 (1,11) |  |
| Papillary craniopharyngioma | 1 (0,09) | 0 (0,00) | 0 (0,00) | 1 (1,11) |  |
| Chordoma, NOS | 1 (0,09) | 1 (0,15) | 0 (0,00) | 0 (0,00) |  |
| Chordoid chordoma | 1 (0,09) | 1 (0,15) | 0 (0,00) | 0 (0,00) |  |
| Papillary tumor of the pineal region | 1 (0,09) | 1 (0,15) | 0 (0,00) | 0 (0,00) |  |
| Protoplasmic astrocytoma | 1 (0,09) | 1 (0,15) | 0 (0,00) | 0 (0,00) |  |
| Primitive neuroectodermal tumor | 1 (0,09) | 1 (0,15) | 0 (0,00) | 0 (0,00) |  |
| Neuroblastoma, NOS | 1 (0,09) | 1 (0,15) | 0 (0,00) | 0 (0,00) |  |
| Anaplastic ganglioglioma | 1 (0,09) | 1 (0,15) | 0 (0,00) | 0 (0,00) |  |
| Hemangioblastic meningioma | 1 (0,09) | 0 (0,00) | 1 (0,28) | 0 (0,00) |  |
| Malignant peripheral nerve sheath tumor | 1 (0,09) | 1 (0,15) | 0 (0,00) | 0 (0,00) |  |
| Hodgkin lymphoma with nodular sclerosis | 1 (0,09) | 1 (0,15) | 0 (0,00) | 0 (0,00) |  |

† χ² test

Sup table 4
**Vital status**

| **Characteristics** | **Total n=1126**  **(100%)** | **Dead n=** **761**  **(67.58 %)** | **Alive n=360**  **(31,97%)** | **Unknown n=5**  **(0,44%)** | **p-value** |
| --- | --- | --- | --- | --- | --- |
| **Sex** |  |  |  |  |  |
| Female | 636 (56.48) | 396 (52.04) | 237 (65.83) | 3 (60.00) | <0.001† |
| Male | 490 (43.52) | 365 (47.96) | 123 (34.17) | 2 (40.00) |  |
| **Tumor behavior** | | |  |  |  |
| Malignant | 676 (60,04%) | 575 (75.56) | 99 (27.50) | 2 (40.00) | 0.011† |
| Benign | 360 (31,97%) | 127 (16.69) | 230 (63.89) | 3 (60.00) |  |
| Uncertain | 90 (7,99%) | 59 (7.75) | 31 (8.61) | 0 (0,00) |  |

† Fisher Test

Sup table 5
**Survival and Risk Ratio**

| **Characteristics** | **5-year survival**  **% (95%CI)** | **Median**  **months (95%CI)** | **HR   (95%CI)** | **p-value** |
| --- | --- | --- | --- | --- |
| **Global Survival** | 57,5 (54,4 - 60,5) | - |  |  |
| **Sex** |  |  |  |  |
| Female | 62,7 (58,6 - 66,5) | - | Reference |  |
| Male | 50,6 (45,8 - 55,2) | - | 1,37 (1,14 - 1,66) | 0,001* |
| **Age** |  |  |  |  |
| 18 – 39 years | 66,09 (59,63 - 1,78) | - | Reference |  |
| 40 – 59 years | 63,10 (58,13 - 67,66) | - | 1,13 (0,85 - 1,48) | 0,380 |
| > 60 years | 46,09 (41,04 - 50,99) | 49,48 (44,38 - 54,36) | 1,92 (1,48 - 2,49) | <0,001 |
| **Age by Decades** |  |  |  |  |
| 20 – 29 years | 68 (57,7 - 76,3) | - |  |  |
| 30 – 39 years | 66,6 (57,8 - 74) | - |  |  |
| 40 – 49 years | 70,4 (63,3 - 76,4) | - |  |  |
| 50 – 59 years | 56,5 (49,4 - 62,9) | - |  |  |
| 60 – 69 years | 54 (35,6 - 60,7) | - |  |  |
| 70 – 79 years | 38,4 (30,3 - 46,4) | 40,5 (32,3 - 48,6) |  |  |
| 80 – 89 years | 38,3 (24,6 - 51,8) | 40,4 (26,4 - 53,9) |  |  |
| > 90 years | 0 | - |  |  |
| **Place of residence** |  |  |  |  |
| Bucaramanga | 60,1 (56 - 63,9) | - | Reference |  |
| Floridablanca | 54,9 (48,3 - 61,3) | - | 1,14 (0,91 - 1,44) | 0,245 |
| Piedecuesta | 52,9 (41,8 - 62,8) | - | 1,19 (0,85 - 1,67) | 0,287 |
| Girón | 52,8 (42,9 - 61,8) | - | 1,25 (0,92 - 1,69) | 0,150 |
| **Socioeconomic status** |  |  |  |  |
| Low bass | 46,8 (35,5 - 57,3) | 53,1 (41,6 - 63,4) | Reference |  |
| Low high | 48,1 (41,4 - 54,5) | 52,7 (45,9 - 59) | 0,93 (0,65 - 1,32) | 0,701 |
| Medium Low | 53,1 (47 - 58,9) | - | 0,78 (0,55 - 1,11) | 0,178 |
| Medium High | 49,3 (41,3 - 56,9) | 55,1 (46,9 - 62,5) | 0,86 (0,59 - 1,26) | 0,461 |
| High Low | 53,3 (34,2 - 69,1) | - | 0,78 (0,42 - 1,42) | 0,422 |
| High High | 36 (18,1 - 54,2) | 44 (24,9 - 61,94) | 1,19 (0,67 - 2,12) | 0,548 |
| **Health insurance modality** | |  |  |  |
| Linked | 50,0 (57,8 – 84,49) | - | Reference |  |
| Subsidized | 52,97 (45,86 – 59,58) | - | 0,80  (0,19 - 3,25) | 0,759 |
| Contribution | 58,35 (54,53 -61,96) | - | 0,63 (0,15 - 2,57) | 0,529 |
| Prepaid Medicine | 61,90 (38,08 -78,08) | - | 0,55 (0,11 - 2,62) | 0,459 |
| Special | 65,50 (51,52 – 71,67) |  | 0,57 (0,13 - 2,39) | 0,447 |
| Particular | 0 |  | 3,39 (0,3 - 37,49) | 0,319 |
| Unknown | 56,52 (34,32 – 73,76) |  | 0,75 (0,16 - 3,43) | 0,714 |
| **Tumor behavior** | |  |  |  |
| Benign | 85,5 (81,4 - 88,8) |  | Reference | <0,001 |
| Uncertain | 64,9 (53,1 - 74,4) |  | 2,85 (1,78 - 4,54) |  |
| Malignant | 39,6 (35,6 - 43,6) | 47,5 (43,4 - 51,5) | 5,74 (4,28 - 7,71) |  |
| **Degree of differentiation** |  |  |  |  |
| T cells |  | - | Reference |  |
| Well differentiating | 60,87 (38,27 -77,37) | - | 3,23 (1,59 - 6,57) | 0,001 |
| Moderately differentiating | 66,67 (50,30 -78,72) | - | 2,38 (1,32 - 4,3) | 0,004 |
| Poorly differentiating | 40,63 (23,83 -56,79) | 43,75 (26,26 - 59,81) | 5,87 (3,46 - 9,95) | <0,001 |
| Undifferentiated/Anaplastic | 28,70 (23,80 -35,54) | 35,65 (29,51 -41,83) | 7,46 (5,43 - 10,23) | <0,001 |
| Cell B | 25, (06,01 -50,48) | 33,3 (10,27 – 58,84) | 7,86 (3,86 - 15,98) | <0,001 |
| Unknown | 48,92 (43,36 – 54,23) | 56,35 (50,76 – 61,55) | 4,63 (3,38 - 6,35) | <0,001 |
| **CNS tumor Location** |  |  |  |  |
| Meninges | 81,7 (77,1 - 85,5) | - | 0,23 (0,17 - 0,3) | <0,001 |
| Other Endocrine Glands | 77,4 (65,8 - 85,5) | - | 0,42 (0,21 - 0,82) | 0,011 |
| Spinal cord and cranial nerves | 67,8 (47,3 - 81,8) | - | 0,28 (0,17 - 0,46) | <0,001 |
| Brain | 41,1 (37,1 - 45,1) | 48,3 (44,2 - 52,2) | Reference |  |
| **Benign Neoplasms** |  |  |  |  |
| Meningiomas | 86,1 (81,4 - 89,5) | - | Referencia |  |
| Other Benign CNS Tumors | 85,71 (53,9 - 96,2) | - | 1,01 (0,24 - 4,2) | 0,968 |
| Pituitary adenoma | 82,6 (69,3 - 90,5) | - | 1,22 (0,59 - 2,52) | 0,585 |
| Ependimomas | 60 (38,45 - 76,11) | - |  |  |
| Prolactinoma | 0 ( - ) |  |  |  |
| **Malignancies** |  |  |  |  |
| Teratoma | 100 ( - ) | - |  |  |
| Peripheral nerve tumors | 85,71 (53,94 - 96,22) | - |  |  |
| Malignant meningiomas | 80 (55,1 - 91,8) | - |  |  |
| Medulloblastoma | 66,67 (28,17 - 87,83) | - |  |  |
| Choroid plexus tumors | 66,67 (5,41 - 94,52) | - |  |  |
| Gliomas | 60 (38,45 - 76,11) | - |  |  |
| Ependymomas | 52,63 (28,72 - 71,88) | - |  |  |
| Other malignant CNS tumors | 52,63 (43,82 - 60,7) | - | 4,07 (2,73 - 6,05) | <0,001 |
| Oligodendroglioma | 51,85 (31,91 - 68,55) | - |  |  |
| Astrocytomas | 51,6 (44,2 - 58,5) | - | 4,14 (2,85 - 6,014) | <0,001 |
| Lymphomas | 36,84 (16,52 - 57,48) | 42,11 (20,37 - 62,49) | - |  |
| Tumor of uncertain malignant behavior | 34,18 (23,99 - 44,6) | 39,64 (28,53 - 49,78) | 7,98 (5,28 - 12,06) | <0,001 |
| Glioneuronal tumors | 25 (0,89 - 66,53) | 25 (0,89 - 66,53) | - |  |
| Glioblastoma | 20,86 (15,36 - 26,93) | 24,06 (18,21 - 30,38) | 9,64 (6,78 - 13,72) | <0,001 |
| Pineal tumor | 0 | - | - |  |
| Neoplasms of uncertain behavior |  |  |  |  |
| Hemangiomas | 82,3 (54,71 - 94,94) | - | - |  |
| Meningioma of uncertain behavior | 80 (55,11 - 91,98) | - | 1,42 (0,5 - 3,97) | 0,503 |
| Other CNS tumors of uncertain behavior | 70,27 (52,78 - 82,3) | - | 2,33 (1,19 - 4,55) | 0,013 |
| Craniopharyngioma | 63,64 (29,69 - 84,52) | - |  |  |
| Ependymomas | 60 (38,45 - 76,11) | - | - |  |
| Tumor of uncertain behavior (unspecified) | 52,63 (28,72 - 71,88) | - | 6,08 (3,19 - 11,6) | <0,001 |

Sup Figure 2
**Survival and Risk Ratio**


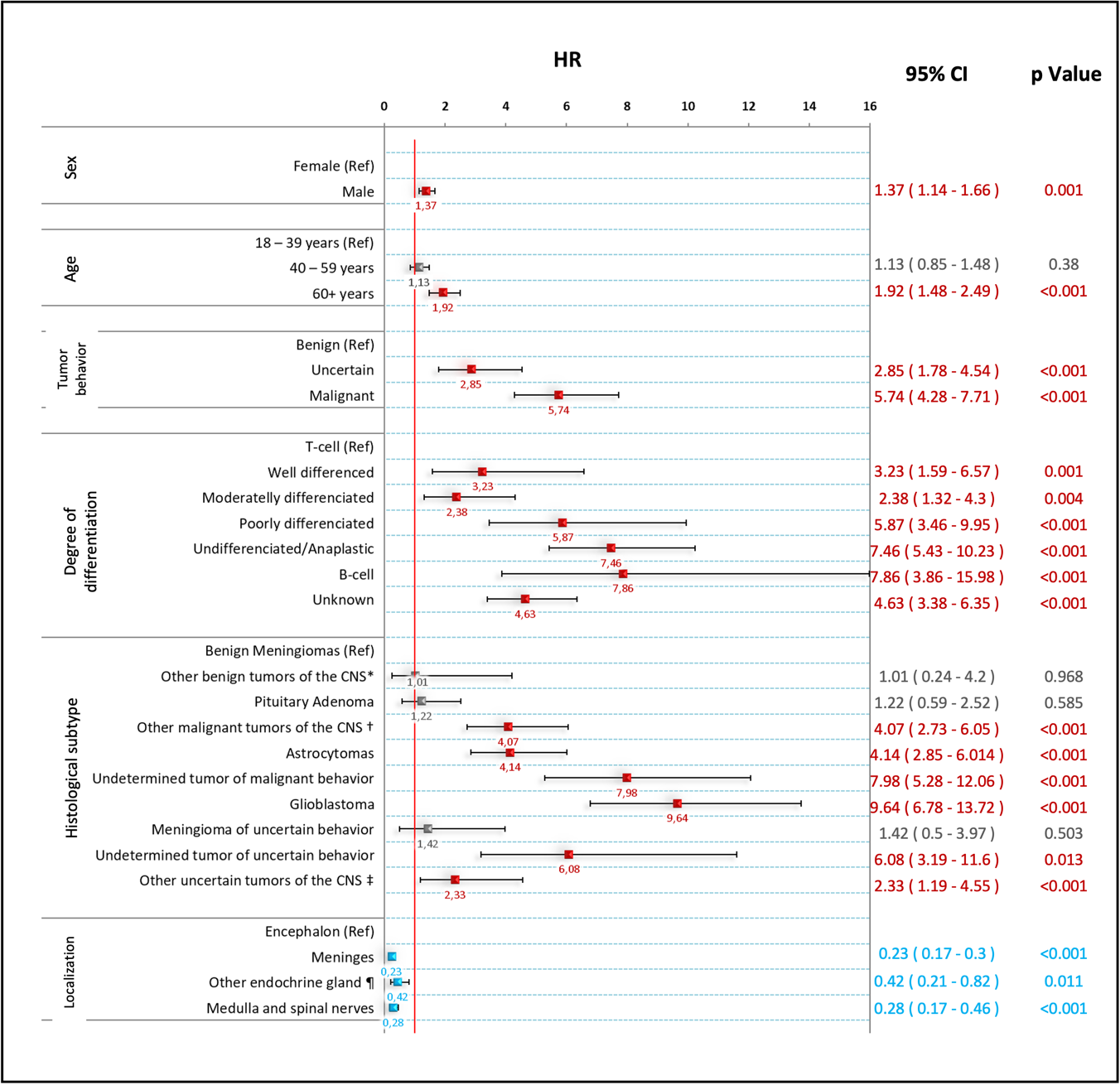

Supplement: Supplementary file 1 — Supplementary Material 1 [file 11060_2025_5313_MOESM1_ESM.docx]
